# *Spironucleus muris* and *Eperythrozoon coccoides* in Rodents from Northwestern Iran: Rare Infections

**Published:** 2018-12-25

**Authors:** Soudabeh Heidari, Mehdi Mohebali, Zabihollah Zarei, Mehdi Nateghpour, Afsaneh Motevalli-Haghi

**Affiliations:** 1 Department of Medical Parasitology and Mycology, School of Public Health, Tehran University of Medical Sciences, Tehran, Iran; 2 Center for Research of Endemic Parasites of Iran, Tehran University of Medical Sciences, Tehran, Iran

**Keywords:** *Spironucleus muris*, *Eperythrozoon coccoides*, Rodents, Iran

## Abstract

**Background:**

Rodents perform a crucial role in dispersal of zoonosis causes globally. We aimed to investigation about infection levels of parasitic agents in rodents’ population in Meshkinshahr areas, northwest of Iran from Apr to Sep 2014.

**Methods:**

Two hundred four rodents were trapped and anaesthetized. A sample of blood was collected via cardiopuncture from each one. Thin and thick blood smears were prepared and stained with Giemsa. All stained smear were examined under light microscopy with high magnification by two expert microscopists. Every suspected unicellular observed were measured microscopically and compared with key references to diagnose.

**Results:**

Captured rodents were identified as three genera including *Meriones persicus*, *Mus musculus*, *Cricetulus migraturius*. Protozoa identified in this study were included of *Spironucleus muris* and *Eperythrozoon coccoides,* these parasites were observed in blood smear of 0.98% of rodents. *S. muris* and *E. coccoides* were seen in *M. musculus* and *C. migraturius*, respectively.

**Conclusion:**

The present study increases awareness about Eperythrozoonosis in rodents and its potential transmission to domestic animals and even to human in rural districts in Iran. Moreover, the attack of *Spironucleus* on the mucus of colon and its systemic risk was confirmed.

## Introduction

Rodentia are the largest group of mammals worldwide. They are found in vast numbers on all continents except Antarctica. Rodents play important roles as reservoirs and carriers of diseases agent such as leishmaniasis, plague, leptospirosis, salmonellosis, rat bite fever, dermatophytosis, Sporotrichosis, murine typhus, trichinellosis, cestodes and trematodes infections, toxoplasmosis, relapsing fever (1).

Blood parasites in rodent such as *Trypanosoma lewisi*, *Leishmania* spp., *Plasmodium berghei*, *Babesia microti*, *Eperythrozoon coccoid*es, *Haemobartonella muris* are important because they are transmitted to humans by ectoparasites of rodents (1).

*Spironucleus muris* (formerly *Hexamita muris*) is an opportunistic pathogen of several rodent species including rat, mice, golden hamster and European hamster. This flagellated protozoan usually inhabits in the crypts of Lieberkuhn in the small intestine after ingesting parasitic cysts and may cause an acute or choronic form of disease. The organisms invade the lamina propria of the intestinal villi in immunocompromised animals and can disseminate systemically through the lymphatics or vasculature. Circulating parasite is visible in the peripheral blood of an infected animal (2–4).

*Eperythrozoon coccoides* is blood parasite in laboratory and wild mice that causes a mild haemolytic anaemia. *Eperythrozoon coccoides* was first identified in laboratory mice in Germany in 1928. Wild mice are natural hosts and rats and rabbits have been experimentally infected. *Eperythrozoon coccoides* was classified into the group of haemotropic mycoplasmas (haemoplasmas) (5–7). Eperythrozoonosis is a zoonotic disease (transmissible from animals to humans). The first recognized human case of eperythrozoonosis was reported in 1986 worldwide (8).

The disease may manifest with fever, hemolytic anemia, lethargy, jaundice, swollen lymph nodes of the neck, leucopenia, neutropenia, thrombocytopenia, splenomegaly and lymphadenopathy, acidosis (9–11). The most important way for the transmission of *E. coccoides* is blood-feeding arthropod vector such as adults and nymphs of the lice *Polypax spinulosa* and *P. serrate*, this transmission is mechanical (12).

We aimed to investigate the diversity and infection levels of parasitaemia in rodent population from Meshkinshahr District.

## Materials and Methods

### Study area

**Fig. 1.**
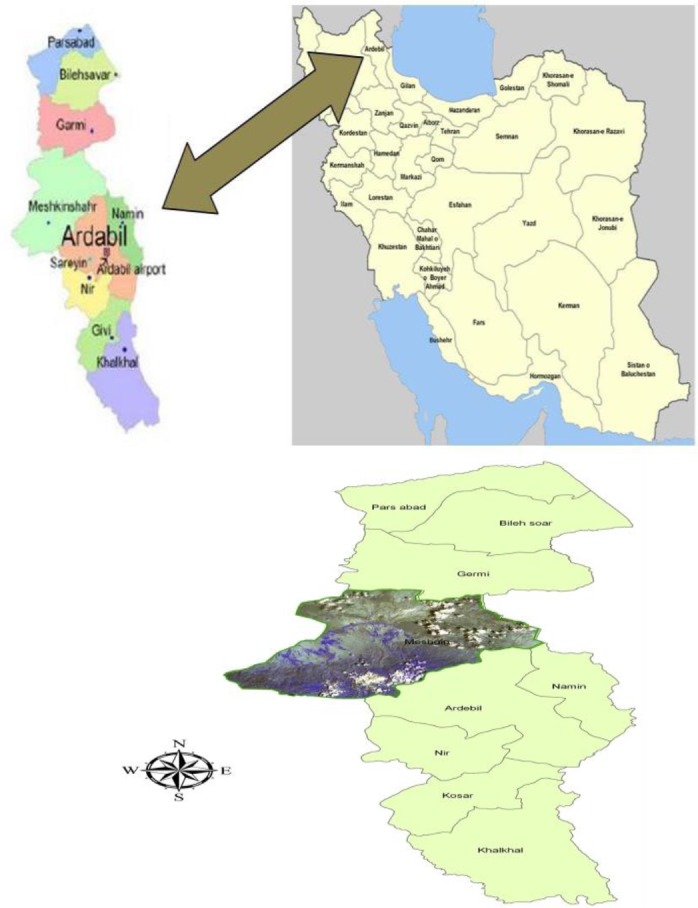
Map of Ardebil Province in Iran (up). The geographical location of collected samples Meshkinshahr in Ardebil (down)

Meshkinshahr City located in Ardebil Province in the northwest of Iran (38°23′56″N 47°40′55″E) (Fig. 1), is situated at an altitude of 1830m above sea level and the average temperature of city is between 22.4 and 2.4 °C. The weather of the city and the district is moderate mountainous. It is limited from the north to the Moghan City and from the west to the Ahar City and from south to the Sabalan high mountain and from the east to Ardebil Province and from the northeast to the Republic of Azerbaijan.

### Sample collection and Parasitological study

Two hundred four rodents (117 *Meriones persicus*, 63 *Mus musculus*, 24 *Cricetulus migraturius* or grey hamsters) were trapped alive from Meshkinshahr Ardebil Province, Iran, between Apr to Sep 2014. Trapped rodents were anaesthetized by placing cotton wool soaked with chloroform. Blood was collected from the heart using a needle and syringe. Thin and thick blood smears were prepared with a drop of blood. Thin blood smear was fixed with methanol. Slides were stained with Giemsa stain and examined under light microscopy at 400x× magnification for parasites screening and 1000× magnification under oil immersion for identification.

### Analysis

Protozoa were microscopically measured and compared with key references (13, 14).

### Ethical consideration

This study was approved by the Research Ethical Review Committee of Tehran University of Medical Sciences, Tehran, Iran (no: 22943).

## Results

**Fig. 2.**
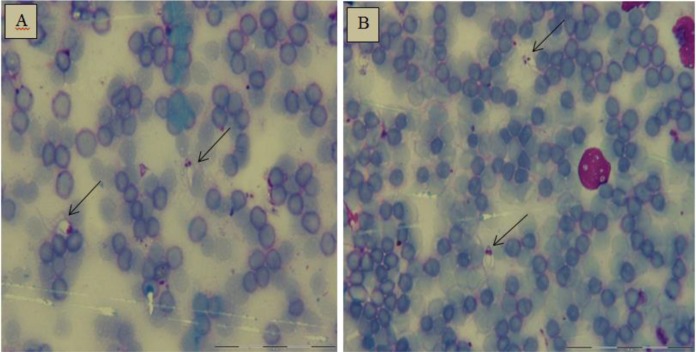
*Spironucleus muris* in a blood smear of *Mus musculus*. Giemsa stain (a and b). Magnification, 1000× (Original)

Two hundred four rodents include 3 genera (117 *M. persicus*, 63 *M. musculus*, 24 *C. migraturius* (grey hamsters) were collected from Meshkinshahr, Ardabil Province. Protozoa identified in this study were included of *S. muris* and *E. coccoides*, these parasites were observed in blood smear of 0.98% of rodents. *Spironucleus muris* was observed in blood smear of one rodent (1.58% of *M. musculus*) (Fig. 2). This organism was ovoid shape approximately 2–3×7–9μm and was bilaterally symmetrical with two nuclei, 6 anterior and 2 posterior flagella (13, 15). In addition, this organism existed in the feces of this rodent.

Moreover, *E. coccoides* was microscopically detected in one rodent (4.16% of hamsters).

**Fig. 3.**
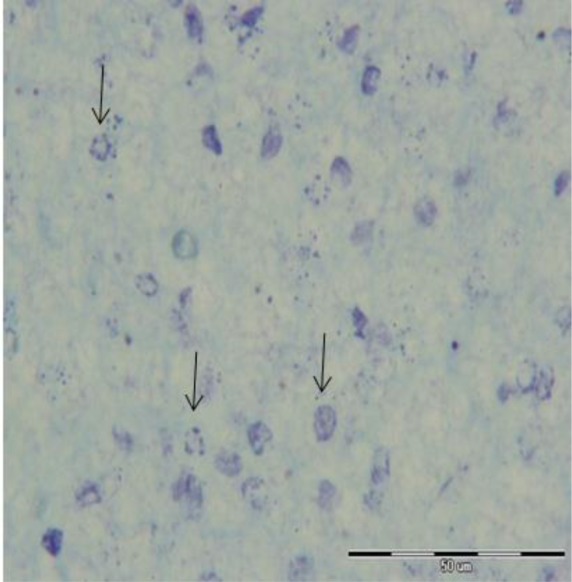
*Eperythrozoon coccoides* in thick blood smear of hamester. Giemsa stain. Magnification, 1000× (Original)

Cocci-shaped objects in large numbers on the surface of red blood cells and red-purple color with size 0.5–3μm (14) were observed in blood smear of this rodent (Fig. 3).

## Discussion

We aimed to determine the diversity of blood parasitaemia in rodent population from Meshkinshahr, Ardabil. Our findings showed presence of two kinds of protozoan parasite including *S. muris* and *E. coccoides.* Previously, *S. muris* has been commonly seen in laboratory rats and mice (16).

The current prevalence is unknown and probably is still high. In a study in Brazil conducted on 344 mice and 111 laboratory rats, 46.2% colonies of mice and 85.7% of rat’s colonies were infected with *S. muris* (17). In another study, 15 mice colonies and 10 rat colonies were collected from 18 laboratories and showed that 90% of rats and 80% of mice were infected with *S. muris* (18). Moreover, in Iraq, 8% of rats were positive for *S. muris* infection (19). Furthermore, a 6.3% of the infection was found in Bandar Abbas, Iran (20). *Spironucleus muris* were detected in fecal samples, indicating the presence of infection in the intestines.

In addition, infection in immunocompetent and adult mice is usually subclinical. However, infection in athymic (*nu/nu*), young and immunocompromised mice characterized clinically by weight loss, enteritis, hunched posture, rough hair coat, hair lacks shine, lethargy, distention of the abdomen, depression, diarrhea and death (3). Trophozoites can cause degeneration enterocytes and shortening of microvilli on the crypt epithelium and increase in crypt depth (2, 21).

Histologically, the formation of cysts was lead to dilation Lieberkuhn glands with an inflammatory reaction in the lamina propria and the sloughing of the epithelium. The main pathological changes had happened in the duodenum, and then in the jejunum and ileum. In acute infections, the lumen of the small intestine has a large number of flagellated organisms that tend to invade the lamina propria of intestinal villi and filling glands. In chronic infection, dilated lymph glands were seen which were similar to the cyst and were filled with inflammatory cells, cellular debris, mucoid material and cystic organisms. Such clinical symptoms has been previously explained (22, 23). Associated pathological findings with the presence of parasite such as pyogranulomatous pneumonia, colitis, lymphadenitis and multifocal abdominal abscess has been previously reported in two immunosuppressed monkeys suffering from systematic *Spironucleus* infection (24).

*S. muris* is an opportunistic pathogen that feeds the intestinal bacteria. Some stress or debilitating factors such as lack of the thymus is necessary for disease (13, 25). *Spironucleus muris* increase the mortality of treated mice with cadmium, as well as is associated with changes in immune function of macrophages in mice and immune responses. Young animals are more sensitive, older animals are not at risk of disease and spontaneously recover mice with history of disease may be resistant to re-infection (26–28). Co-infection with *B. microti*, *P. berghei*, and *P. yoelii*, has been reported with reducing of the trophozoite production in *S. muris* (29).

Furthermore, microvilli damage, loss of height of microvilli, increase in crypt depth, hyperplasia of crypt, abscesses in crypts and atrophy of villis have been reported in mice with *S. muris* (21, 28). This organism is likely to be systemic through the attack to the mucus of colon. Large lesions in the lymph nodes are most likely to indicate the diffusion of parasites from the liver through lymphatic vessels as compared to blood circulation. However, the vessels diffusion cannot be disproved. After enterocytes destruction and necrosis, trophozoites of *S. muris* pass from the intestinal barrier and enter the blood stream in these areas (24, 30). In the present study, one out of 204 (0.49%) rodents was infected with *S. muris* in the intestinal contents and blood. It may be interpreted that the parasites had passed from the intestinal barrier and entered to the bloodstream. Numerous parasites were observed in peripheral blood.

In this study, *E. coccoides* was another organism detected and is a parasitic bacteria that attach to the surface of erythrocytes in mouse which can induce erythrocytic damage. This organism was previously classified as protozoa but in 2005, based on phylogenetic evidence and 16S rRNA gene sequences was classified in haemotropic mycoplasmas and family of mycoplasmataceae (7). *Eperythrozoon coccoides* is mechanically transmitted to humans by arthropods and it is important because human infection have been reported. Infection is more common in farmers and veterinarians who are in close contact with domestic animals. Congenital transmission has also been reported (31). Infected people may be asymptomatic or symptoms, including fever, severe hemolytic anemia and jaundice, especially in infants. Pregnant women and infants are more vulnerable than others and show more severe symptoms.

In Iran, there is no report of *E. coccoides* in animals and humans. In this study, one out of 204 rodents (0.49%) was infected with *E. coccoides.* A molecular and microscopic study of blood in China indicated that *E. coccoides* were causative agent of anemia in a person who has a long history of anemia (9). In a meta-analysis and systematic study, the rate of infection with *Eperythrozoon* species in the population of Chinese has been investigated. Overall, 14951 out of 52433 cases were infected. According to the seasons and the geographical areas, the infection rate varied from 0% to 97.29% (32).

## Conclusion

The present study increases awareness about Eperythrozoonosis in rodents and its potential transmission to domestic animals and even to human in rural districts in Iran. Moreover, information obtained from this study confirmed the attack of *Spironucleus* on the mucus of colon and its systemic risk.
